# Do methadone and buprenorphine have the same impact on psychopathological symptoms of heroin addicts?

**DOI:** 10.1186/1744-859X-10-17

**Published:** 2011-05-15

**Authors:** Angelo Giovanni Icro Maremmani, Luca Rovai, Pier Paolo Pani, Matteo Pacini, Francesco Lamanna, Fabio Rugani, Elisa Schiavi, Liliana Dell'Osso, Icro Maremmani

**Affiliations:** 1 'Vincent P. Dole' Dual Diagnosis Unit, Santa Chiara University Hospital, Department of Psychiatry, NPB, University of Pisa, Pisa, Italy; 2 AU-CNS, 'From Science to Public Policy' Association, Pietrasanta, Lucca, Italy; 3 'G. De Lisio', Institute of Behavioral Sciences Pisa, Pisa, Italy; 4 Sardinia Health and Social Administration, Sardinia Autonomous Region, Cagliari, Italy; 5 Ser.T (Drug Addiction Unit), Pisa, Italy

## Abstract

**Background:**

The idea that the impact of opioid agonist treatment is influenced by the psychopathological profile of heroin addicts has not yet been investigated, and is based on the concept of a specific therapeutic action displayed by opioid agents on psychopathological symptoms. In the present report we compared the effects of buprenorphine and methadone on the psychopathological symptoms of 213 patients (106 on buprenorphine and 107 on methadone) in a follow-up study lasting 12 months.

**Methods:**

Drug addiction history was collected by means of the Drug Addiction History Rating Scale (DAH-RS) and psychopathological features were collected by means of the Symptom Checklist-90 (SCL-90), using a special five-factor solution. Toxicological urinalyses were carried out for each patient during the treatment period.

**Results:**

No statistically significant differences were detected in psychopathological symptoms, including 'worthlessness-being trapped', 'somatization', and 'panic-anxiety'. Methadone proved to be more effective on patients characterized by 'sensitivity-psychoticism', whereas buprenorphine was more effective on patients displaying a 'violence-suicide' symptomatology.

**Conclusions:**

Heroin-dependent patients with psychiatric comorbidities may benefit from opioid agonist treatment not only because it targets their addictive problem, but also, precisely due to this, because it is effective against their mental disorder too.

## Background

While psychiatric comorbidity has been shown to have a negative impact on the outcome of opioid use disorders [1-9], studies carried out in the context of Methadone Maintenance Treatment Programs (MMTPs) to evaluate outcomes strictly linked with methadone efficacy have not demonstrated any such negative influence [10-14].

The complex nature of psychopathology in substance abuse disorders (SUDs), is particularly difficult to assess at the moment of admission to treatment, when the heterogeneity of the psychological/psychiatric conditions displayed impairs the attribution of symptoms to psychiatric conditions preceding the initial use of substances, to the effects of heroin and/or other substances, to neurobiological addictive processes, or to psychosocial stress associated with addictive behavior [15-18]. On these bases a unitary perspective has been proposed, foreseeing the inclusion of symptoms of anxiety, mood and impulse-control domains in the psychopathology of addiction, but also taking into account symptoms and syndromes that are under the threshold for the definition of an additional mental disorder, although they may have a strong effect on the everyday life of patients and may frequently require intervention [19,20].

This approach is consistent with the often-found tendency in the field of addiction to evaluate the impact of psychopathology on the outcome of a treatment in terms of the severity of the psychological/psychiatric problems involved through the use of rating scales and interviews such as the Symptom Checklist-90 (SCL-90) and Anxiety Sensitivity Index (ASI), rather than in terms of formal psychiatric diagnoses [21-25].

Recently, using the SCL-90, we studied the psychopathological dimensions of 1,055 patients with heroin addiction (884 males and 171 females) aged between 16 and 59 years at the beginning of treatment, and their relationship to age, sex and duration of dependence. We found five subgroups of patients characterized by (1) depressive symptomatology with prominent feelings of worthlessness-being trapped or caught, (2) somatization symptoms, (3) interpersonal sensitivity and psychotic symptoms, (4) panic symptomatology, and (5) violence and self-aggression. These groups were not correlated with sex or duration of dependence. Younger patients with heroin addiction were more strongly represented in prominent violence-suicide, sensitivity and panic-anxiety symptomatology groups. Older patients were more strongly represented in prominent somatization and worthlessness-being trapped symptomatology groups [26].

Therefore, we wondered if methadone and buprenorphine have the same impact on the psychopathological dimensions mentioned above.

In a previous study we evaluated the efficacy of buprenorphine and methadone on psychopathological symptoms according to a standard SCL-90 nine-factor structure [27]. We treated 213 patients (106 of these on buprenorphine and 107 on methadone) in an open study, following patients between months 3-12 of their treatment; those who left the program before the end of their third month of treatment were excluded from the study sample. The results of this study showed statistically significant improvements in opioid use, psychiatric symptomatology and quality of life between months 3-12 for both medications [24].

In the present study we compared the effects of buprenorphine and methadone on the psychopathological symptoms of these same patients after re-evaluation on the basis of our new five-factor SCL-90 structure.

## Methods

### Sample

The sample comprised 213 heroin-dependent patients selected according to *Diagnostic and Statistical Manual of Mental Disorders*, 4th edition, text revision (DSM-IV-TR) criteria [28]: their mean age was 31 (SD 6), 176 (82.6%) were males, 130 (61.0%) were single, 135 (63.4%) had a low educational level (≤8 years), 81 (38%) were unemployed and 6 (2.8%) were receiving welfare benefits. In all, 106 patients were being treated with buprenorphine and 107 with methadone. For further details, please see Maremmani *et al. *[24].

On the basis of the highest z scores obtained on the five SCL-90 factors (dominant SCL-90 factor) (see Instruments section below) subjects were assigned to five mutually exclusive groups. Six subjects (2.8%) had missing data. The group whose dominant factor was 'worthlessness-being trapped' comprised 33 subjects (15.6%), the group with 'somatization' as its dominant factor was made up of 43 subjects (20.3%), the group showing 'sensitivity-psychoticism' as its dominant factor included 31 subjects (14.6%), the group identified by 'panic-anxiety' as its dominant factor numbered 66 subjects (30.3%), and the group whose dominant factor was 'violence-suicide' profiled a cluster of 39 subjects (17.9%). These five groups were sufficiently distinct, and did not show any significant overlap. All these patients showed positive scores in their dominant factors only, alongside negative scores in all the others; the only exception being a small number of patients whose dominant factor was 'worthlessness-being trapped', who recorded a positive score for the 'sensitivity psychoticism' factor (mean ± SD = 0.06 ± 0.5) This finding was confirmed by the discriminant analysis, which indicated a percentage of correctly classified 'grouped' cases as high as 90.1%.

## Instruments

### Drug Addiction History Rating Scale (DAH-RS)

The DAH-RS [29] is a multiscale questionnaire comprising the following categories: sociodemographic information, physical health, mental health, substances abused, treatment history, social adjustment and environmental factors. The questionnaire rates ten items: physical problems, mental problems, substance abuse, previous treatment, associated treatments, employment status, family situation, sexual problems, socialization and leisure time, legal problems. (The specific clinical variables addressed are: hepatic, vascular, hemolymphatic, gastrointestinal, sexual, dental pathology, HIV serum status, memory disorders, anxiety disorders, mood disorders, aggressiveness, thought disorders, perception disorders, awareness of illness; employment, family, sex, socialization and leisure time, legal problems; use of alcohol, opiates, central nervous system (CNS) depressants, CNS stimulants, hallucinogens, phencyclidine, cannabis, inhalants, polysubstance abuse, frequency of drug use, pattern of use, previous treatments and current treatments). Items are constructed in order to obtain dichotomous answers (yes/no).

### SCL-90

The SCL-90 [27] is an inventory composed of 90 items, with a point scale ranging from 0 to 5, to allow assessment of intensity. The items are grouped into five factors related to different psychopathological dimensions: worthlessness-being trapped, somatization, sensitivity-psychoticism, panic-anxiety and violence-suicide. The five-factor solution is based on an exploratory factor analysis we performed on the 90 SCL items. This analysis involved 1,055 patients [26]. The ratio of patients/items (11:1) was high enough to authorize this analysis, as it is higher than the recommended 10:1 ratio. Factors were extracted by using a main component analysis (principal component analysis (PCA) type 2) and then rotating this orthogonally to achieve a simple structure. This simplification is equivalent to maximizing the variance of the squared loading in each column. To limit the factor number, the criterion used was an eigenvalue >1.5. Items loading with absolute values >0.40 were used to describe the factors. This procedure makes it possible to minimize the crossloadings of items on factors. In order to make factor scores comparable, they can be standardized into z scores. All subjects can be assigned to one of the five different subtypes on the basis of the highest factor score achieved (dominant SCL-90 factor). This procedure allows the classification of subjects on the basis of their dominant symptomatological cluster. In this way it is possible to solve the problem of identifying a cut-off point for the inclusion of patients in the different clusters identified.

### Urinalysis

The toxicological urinalyses were expressed using two indices, PCC (PerCent 'Clean') and TEC (out of Total Executed percent 'Clean'). PCC expresses the percentage ratio of urinalyses proving negative for the presence of morphine and the total number of urinalyses carried out for each patient during the period of treatment. TEC is the percentage ratio between the number of urinalyses that proved to be negative for the presence of morphine and the number of urine analyses that the protocol has envisaged throughout the process. In this case, the reference number was 37 (the maximum number of urine samples per patient). PCC tends to give preference to patients who remain 'opiate free', but who terminate the study in advance for reasons not correlated with the study (for example, imprisonment). TEC additionally considers how long the patient remains in the protocol, and gives less precedence to these patients. These two indices represent the two extremes, but results tend to balance out. With regard to these parameters, the comparison between the two groups was made with Student's *t *test.

### Data analysis

Analysis of the results was performed on completion of the 12 months of treatment. Patients belonging to one of the five dominant subgroups and undergoing treatment, with buprenorphine or with methadone, were compared for their retention in treatment. Retention in treatment was analyzed by means of survival analysis and Leu-Desu statistics for comparison between the survival curves. For the purpose of this analysis, 'completed observations' is a term that refers to patients who left the treatment, while 'censored observations' refers to patients who are still in treatment at the end of the 12 month period or have decided to leave the treatment for reasons unrelated to treatment (for example, patients moving to other towns, imprisonment, and so on). The homogeneity of the population samples treated with buprenorphine or methadone according to SCL-dominant groups was tested by means of Student's *t *test for continuous variables and χ^2 ^test for categorical variables. We used the statistical routines in SPSS V.4.0 (SPSS, Chicago, IL, USA).

## Results

At 12 months (Table 1) no statistically significant difference was observed regarding subjects belonging to the 'worthlessness-being trapped' dominant group and treated with methadone or buprenorphine. Similarly, no statistically significant differences were observed for patients belonging to the 'somatization', and 'panic-anxiety' dominant groups.

**Table 1:** Survival in treatment of buprenorphine-treated or methadone-treated heroin-dependent patients according to dominant psychopathological groups

	N	CEN*	%	*P *value
Independently of psychopathology				

Buprenorphine	108	88	81.48	

Methadone	104	84	80.77	0.94

Worthlessness-being trapped				

Buprenorphine	18	14	77.78	

Methadone	15	9	60.00	0.39

Somatization				

Buprenorphine	24	20	83.33	

Methadone	19	17	89.47	0.58

Sensitivity-psychoticism				

Buprenorphine	15	8	53.33	

Methadone	16	14	87.50	0.03

Panic-anxiety				

Buprenorphine	29	25	86.21	

Methadone	37	32	86.49	0.98

Violence-suicide				

Buprenorphine	19	19	100.00	

Methadone	20	14	70.00	0.01

* censored

Regarding the 'sensitivity-psychoticism' dominant group, 14 (87.5%) out of 16 patients in treatment with methadone were still in treatment. During the same period, only 8 (53.3%) out of 15 patients in treatment with buprenorphine were still in treatment. This difference was statistically significant. Patients treated with buprenorphine or methadone did not differ significantly in rates for gender, education, civil status, presence of somatic comorbidity, psychiatric comorbidity, baseline household major problems, sexual major problems, social-leisure major problems, legal problems or polyabuse. No significant differences were observed either in age, age at first use of substances, age at dependence onset, dependence duration or age at first treatment. During the follow-up period no statistically significant differences were observed regarding urinalyses for heroin or cocaine metabolites. More unemployed patients with work major problems and with past unsuccessful treatments were present in the methadone group (see Table 2).

**Table 2:** Demographic and clinical characteristics of the sensitivity-psychoticism dominant groups according to treatment

	Buprenorphine (N = 15)	Methadone, (N = 16)		*P *value
	N (%)	N (%)	χ^2^	

Gender (males)	13 (86.7)	14 (87.5)	0.00	0.944

Work:			7.72	0.052

Student	0 (0.0)	1 (6.3)		

Blue collar	2 (20.0)	3 (18.8)		

White collar	11 (73.3)	5 (31.3)		

Unemployed	1 (6.7)	7 (43.8)		

Education: >8 years	4 (26.7)	5 (31.3)	0.07	0.778

Civil status: single	13 (86.7)	12 (75.0)	0.67	0.411

Somatic comorbidity	10 (66.7)	13 (81.3)	0.85	0.350

Psychiatric comorbidity	10 (66.7)	14 (93.3)	3.33	0.060

Work major problems	0 (0.0)	7 (46.7)	9.1	0.002

Household major problems	14 (93.3)	13 (81.3)	1.00	0.315

Sexual major problems	12 (80.0)	13 (81.3)	0.00	0.929

Social-leisure major problems	11 (73.3)	12 (75.0)	0.01	0.915

Legal problems	2 (13.3)	6 (37.3)	2.36	0.124

Polyabuse	9 (60.0)	10 (62.5)	0.02	0.886

Past unsuccessful treatments	8 (53.3)	16 (100.0)	9.64	0.001

	Mean ± SD	Mean ± SD	T*	

Age	27 ± 5	30 ± 4	-1.90	0.067

Age at first use, years	18 ± 5	19 ± 5	-0.75	0.463

Age at dependence onset, years	20 ± 5	23 ± 5	-1.09	0.284

Dependence duration, months	53 ± 40	75 ± 46	-1.36	0.186

Age at first treatment, years	22 ± 5	25 ± 4	-1.54	0.136

Heroin PCC	89.16 ± 27.5	83.96 ± 17.9	0.62	0.542

Heroin TEC	21.84 ± 13.9	25.59 ± 15.4	-0.70	0.490

Cocaine PCC	94.16 ± 13.3	85.83 ± 16.3	1.56	0.130

Cocaine TEC	22.88 ± 12.6	23.60 ± 16.5	-0.12	0.902

* Student T-test; PCC = Percent 'clean'; TEC = Total Executed 'Clean'

Considering the 'violence-suicide' dominant group, all (n = 19) patients treated with buprenorphine were still in treatment. During the same period, 14 (70.0%) out of 20 patients in treatment with methadone were still in treatment. This difference was statistically significant. Patients treated with buprenorphine or methadone did not differ significantly in rates of employment, education, civil status, presence of somatic comorbidity, psychiatric comorbidity, baseline work major problems, household major problems, sexual major problems, legal problems, polyabuse or unsuccessful treatments in the past. No significant differences were observed either in age, age at first use of substances, age at dependence onset, dependence duration, age at first treatment. During the follow-up period no statistically significant differences were observed regarding urinalyses for heroin or cocaine metabolites. More males and patients with social-leisure major problems were present in the buprenorphine group (see Table 3).

**Table 3:** Demographic and clinical characteristics of the violence-suicide dominant groups according to treatment

	Buprenorphine (N = 19)	Methadone, (N = 20)		*P *value
	N (%)	N (%)	χ^2^	

Gender (males)	18 (94.7)	12 (60.0)	6.62	0.01

Work:			3.56	0.313

Student	3 (15.8)	0 (0.0)		

Blue collar	4 (21.1)	4 (20.0)		

White collar	7 (36.8)	9 (45.0)		

Unemployed	5 (26.3)	7 (35.0)		

Education: >8 years	8 (42.1)	11 (55.0)	0.64	0.42

Civil status: single	11 (57.9)	9 (45.0)	0.64	0.42

Somatic comorbidity	11 (57.9)	12 (60.0)	0.01	0.893

Psychiatric comorbidity	14 (77.8)	16 (84.2)	0.24	0.617

Work major problems	5 (26.3)	8 (42.1)	1.05	0.304

Household major problems	17 (89.5)	17 (89.5)	0	1

Sexual major problems	17 (89.5)	17 (94.4)	0.3	0.579

Social-leisure major problems	16 (84.2)	8 (42.1)	7.23	0.007

Legal problems	7 (36.8)	7 (35.0)	0.01	0.904

Polyabuse	11 (57.9)	15 (75.0)	1.28	0.257

Past unsuccessful treatments	14 (73.7)	18 (90.0)	1.76	0.184

	Mean ± SD	Mean ± SD	T*	

Age	28 ± 7	30 ± 6	-1.13	0.264

Age at first use, years	16 ± 2	18 ± 4	-1.79	0.082

Age at dependence onset, years	18 ± 2	20 ± 4	-1.62	0.116

Dependence duration, months	81 ± 67	124 ± 94	-1.63	0.112

Age at first treatment, years	21 ± 3	24 ± 4	-1.91	0.065

Heroin PCC	92.74 ± 10.7	80.52 ± 27.7	1.83	0.079

Heroin TEC	30.60 ± 19.2	30.58 ± 27.7	0	0.998

Cocaine PCC	87.23 ± 24.8	86.62 ± 19.6	0.08	0.933

Cocaine TEC	30.38 ± 24.3	34.06 ± 29.4	-0.4	0.691

* Student T-test; PCC = Percent 'clean'; TEC = Total Executed 'Clean'

## Discussion

In our sample, the question of whether a patient belonged to one of the 'worthlessness-being trapped', 'somatization' and 'panic-anxiety' dominant groups did not affect survival in treatment. Patients with 'sensitivity-psychoticism' as their predominant characteristics showed a better outcome when treated with methadone. Patients with 'violence-suicide' as their predominant characteristics showed a better outcome when treated with buprenorphine. This occurred despite the fact that methadone-treated sensitivity-psychoticism patients showed a higher frequency of unemployment, of work major problems and of unsuccessful treatments in the past compared with patients possessing the same predominant characteristics who were treated with buprenorphine. Buprenorphine-treated violence-suicide patients were characterized by the male gender and showed a better outcome, despite the presence of social-leisure major problems. In our sample methadone and buprenorphine showed the same effect on heroin dependence (as proved by results for urinalyses that were not statistically different), but did show a different impact on psychopathology when patients were assessed using our new five-factor SCL-90 solution.

The impact of long-acting opioid treatment on the psychopathological profile of heroin addicts has not yet been fully investigated, despite the possibility (reported in the literature) that opioid agents have a specific therapeutic action on psychopathological symptoms.

In the literature, opioid agents have been reported to have a therapeutic effect in a wide range of psychopathological conditions. This is also suggested by the fact that dual diagnosis heroin addicts need higher stabilization dosages (150 mg/day on average) than those without any additional psychiatric disorder (whose average dose is 100 mg/day) [11].

With regard to mood disorders, opiates were used to treat major depression until the 1950s. More recently, consistently with the endorphinergic hypothesis of dysthymic disorders [30] opioid peptides have been considered potential candidates for the development of novel antidepressant treatment [31,32].

On clinical grounds, the efficacy of β-endorphins has been assessed on non-addicted depressed patients [33]. Codeine has been evaluated as a possible therapeutic agent in the treatment of involutional and senile depression [34]. More recently buprenorphine, thanks to its partial agonist activity, bringing with it a reduced risk of dependence and abuse, has turned out to offer an effective therapeutic strategy in depressed patients who are unresponsive to, or intolerant of, conventional antidepressant agents [35-37].

Although opiates are known to produce euphoric states, and spontaneous states of elation are associated with high CNS levels of endorphins, a low incidence of manic states has been reported among heroin addicts. Methadone maintenance has been observed to achieve major mood stabilization in bipolar I patients; this supports the idea that opioid agonists may display an antimanic effect [11,32,38]. The opiate antagonist naloxone has likewise shown antimanic properties probably attributable to its hypothesized negative influence on basal mood, formulated on the basis of observations on addicted or non-addicted patients [39-42].

With regard to anxiety disorders, opioid agents have been reported to display antipanic effects [32]. Consistently with these observations, naltrexone has been shown to elicit anxiety and to induce panic attacks in non-addicted as well as addicted patients [40].

Some authors have hypothesized a direct involvement of opioid neuropeptides in the pathophysiology of psychotic disorders [43]. The antipsychotic effectiveness of opiate agonists [44] is supported by the fact that methadone maintenance is responsible for the prevention of psychotic relapses in individuals with a history of psychotic episodes. In the same subjects, the gradual elimination of methadone was followed by psychotic relapses [45]. The use of methadone has been proposed as a treatment in cases of schizophrenia that have turned out to be resistant to traditional medications, and again in cases of the early development of dyskinesias [46]. Going forward when combined with methadone, low dosages of antipsychotics, such as chlorpromazine, flufenazine and haloperidol are needed to control psychotic symptoms [47-49]. This therapeutic suggestion is in line with the antidopaminergic activity of methadone, as documented by the increase in serum prolactin after its administration [50]. In line with these observations, our heroin-dependent patients with prominently psychopathological sensitivity-psychoticism characteristics showed a better level of retention in treatment when treated with methadone.

A series of studies indicates that opiate agonists are likely to be effective in controlling aggressive behavior in opiate-addicted patients, as confirmed by the fall in levels of aggressiveness which follows adequate methadone treatment [51,52]. Moreover, aggressive symptoms are among the features that may be found in the habit of applying a self-medication theory [53]. In this study buprenorphine showed better results than methadone in patients with prominently aggressive characteristics (in the violence-suicide dominant group).

## Conclusions

The observations reported in the literature and the results of this study suggest that opioid agonists should be reconsidered, as they not only possess an anticraving activity but are also able to act as psychotropic instruments in treating mental illness, with special reference to mood, anxiety and psychotic syndromes. In particular, methadone seems to be more effective on sensitivity-psychoticism aspects, whereas buprenorphine seems to be more effective on aggressive behavior (violence-suicide). As a result, some dual diagnosis patients may benefit from a treatment (methadone or buprenorphine) that not only targets their addictive problem but is also effective on their mental disorder.

## Competing interests

The authors declare that they have no competing interests.

## Authors' contributions

AGIM, LR, PPP and IM conceived the study, participated in its design and coordination, and helped to draft the manuscript. MP, FL, FR, ES and LDO revised the literature and participated in interpretation of data. All authors read and approved the final manuscript.
